# Nasal Obstruction

**Published:** 1899-06

**Authors:** 


**Nasal Obstruction.** By W. J. WALSHAM, M.B. Pp. viii., 256. London: Baillière, Tindall and Cox. 1898.

The author of this work has proceeded on somewhat unusual lines. He begins by working from the known conditions of the parts, such as redness, hardness, swelling, &c., to the unknown disease of which they are signs and symptoms, instead of first describing the disease and then discussing how it may be diagnosed from similar affections. He thus goes through all the diseases which give rise to nasal obstruction, and then finally he gives the treatment necessary for each variety in separate chapters at the end of the book. This method of teaching is frequently made use of in clinical lectures or in oral instruction during clinical practice, but it has many disadvantages when used in text-books. Thus it necessitates a great deal of repetition, it is wearying to the reader, and it separates the remarks on treatment from their natural position immediately after the diagnosis and symptoms and places them by themselves in another part of the book. To these objections with the author's methods we would add unstinting praise for his matter. The descriptions of the conditions giving rise to nasal obstruction are most lucid and admirable. They are clearly defined and well differentiated. The treatment advised is eminently cautious and conservative, and does not partake of that meddlesomeness so common in the present day. All "spoke-shaving" of turbinal bodies is deprecated, and we do not find any mention of that bogey "necrosing ethmoiditis." The author states that, although he has performed a large number of intra-nasal operations, he has never known them to be followed by any septic sequelæ. The most rigid precautions are always taken to work with absolutely aseptic instruments and hands, but the nose having been shown to be practically an aseptic cavity, he never employs antiseptics in the form of washes or douches before operating, and having taken care not to introduce any septic material from without, he leaves the cavity after the operation rigorously alone. When this is done, an aseptic blood-clot forms, and no better antiseptic dressing or preventive for the injured tissue can be found.

